# Microglial autophagy in Alzheimer’s disease and Parkinson’s disease

**DOI:** 10.3389/fnagi.2022.1065183

**Published:** 2023-01-10

**Authors:** Zhifu Wang, Qi Wang, Shihua Li, Xiao-Jiang Li, Weili Yang, Dajian He

**Affiliations:** Guangdong Key Laboratory of Non-human Primate Research, Guangdong-Hongkong-Macau Institute of CNS Regeneration, Jinan University, Guangzhou, China

**Keywords:** neurodegeneration, microglia, autophagy, AD, PD

## Abstract

Alzheimer’s disease (AD) and Parkinson’s disease (PD) are the most common neurodegenerative diseases, characterized by gradual and selective loss of neurons in the central nervous system. They affect more than 50 million people worldwide, and their incidence increases with age. Although most cases of AD and PD are sporadic, some are caused by genetic mutations that are inherited. Both sporadic and familial cases display complex neuropathology and represent the most perplexing neurological disorders. Because of the undefined pathogenesis and complex clinical manifestations, there is still no effective treatment for both AD and PD. Understanding the pathogenesis of these important neurodegenerative diseases is important for developing successful therapies. Increasing evidence suggests that microglial autophagy is associated with the pathogenesis of AD and PD, and its dysfunction has been implicated in disease progression. In this review, we focus on the autophagy function in microglia and its dysfunction in AD and PD disease models in an attempt to help our understanding of the pathogenesis and identifying new therapeutic targets of AD and PD.

## Introduction

Microglia are the resident mononuclear phagocytes in the central nervous system (CNS), belonging to the non-neuronal glial system and supporting neuronal functions. They are originated from the myeloid and broadly distributed throughout the brain and spinal cord (Ginhoux et al., 2013). During development, microglia sculpt immature neuronal circuits by phagocytizing and eliminating synaptic structures (axons and dendritic spines) in a process known as synaptic pruning (Paolicelli et al., 2011; Schafer et al., 2012). In CNS parenchyma, microglia account for 5%–20% of the total glial cell population and 10% of the non-neuronal cells (Lawson et al., 1990; Perry, 1998). They play fundamental roles in the control of immune responses and the maintenance of CNS homeostasis (Perry et al., 2010; Salter and Stevens, 2017). They also carry other exclusive characteristics, such as unique motility with their fine processes, which can scan the whole brain parenchyma every few hours (Davalos et al., 2005; Nimmerjahn et al., 2005).

Under physical conditions, microglia phagocytose synaptic structures to remodel the presynaptic environment and release soluble factors in the mature and aging brains (Tremblay et al., 2011; Su et al., 2016). However, under certain pathological conditions or during aging, this function can go beyond physiological control, resulting in an inflammatory state and the progression of neurodegeneration. Understanding age-related microglia response is important for the cure of the severe neurodegenerative diseases (Fuger et al., 2017; Salter and Stevens, 2017).

Neurodegenerative diseases are progressive disorders, characterized by the age-dependent loss of neuronal structures or functions that leads to neuronal death (Ross and Poirier, 2004). These diseases, including AD and PD, are increasingly being realized to share common cellular and molecular mechanisms. They are featured by selective neuronal vulnerability in specific brain regions and intracellular or extracellular deposits of abnormal proteins in or between neurons and other cells (Wong et al., 2002; Nussbaum and Ellis, 2003; Selkoe, 2003; Ross and Pickart, 2004). Emerging evidence points to the inflammatory responses in the CNS as a major cause and a common feature in neurodegenerative diseases. Pro-inflammatory cytokines are chronically increased in the aging brain (Godbout et al., 2005; Sierra et al., 2007), which is correlated with characteristics of the occurrence of chronic neurodegenerative diseases.

In this review, we summarized the current knowledge of microglial autophagy in the chronic neurodegeneration process and discuss the roles of microglial autophagy in disease pathogenesis and its potential as a new therapeutic target.

## Microglia reaction in neurodegeneration

### Autophagy process in microglia

Aging is the major risk factor for neurodegeneration diseases. Microglia play fundamental roles in aging progress and neurodegenerative disease progression. Although a large body of work about the functional dynamics of microglia during aging has been reported and although phagocytosis is known to be executed mainly by microglia, the elaborated details and the involvement of microglial dysfunction in neurodegenerative diseases remain to be fully investigated (Sierra et al., 2013; Abiega et al., 2016).

Neurons depend largely on autophagy to maintain cellular homeostasis by eliminating pathological protein aggregates, and defect of this process contributes to the pathologies of many neurodegenerative diseases (Marino et al., 2011; Goldsmith et al., 2022). Autophagy is an evolutionarily conserved cellular degradation and recycling process that initiates with the formation of a double membrane structure, associated with the endoplasmic reticulum in mammalian cells. Compromising the autophagy pathway can contribute to neurodegenerative diseases, such as PD, and impaired autophagy process is proved in postmortem brain samples from patients with AD and PD (Anglade et al., 1997; Nixon et al., 2005; van Beek et al., 2018). Although most studies assessing the role of CNS autophagy in aging and neurodegeneration have focused on neurons, emerging findings suggest that autophagy may also vitally be regulated by glial cells (Choi et al., 2020). Recent evidence indicates that specific impairment of noncanonical autophagy in microglia could reduce the capacity of clearance β-amyloid and result in progressive neurodegeneration in a mouse model of AD (Heckmann et al., 2019), which is consistent with previously reported results in autophagy-related gene 7 (*atg7*) knockout mice (Cho et al., 2014). In PD mouse model, a*tg5* knockout microglia can aggravate neuroinflammation and dopaminergic neuron loss in the substantia nigra (SN; Tu et al., 2021). Therefore, targeting microglial autophagy in these diseases has a proposed therapeutic potential.

## Key autophagy regulators and pathways in microglia

In general, microglia participate in autophagy by phagocytosis in the CNS. Because of the critical role of autophagy in protein and organelle quality control (Mizushima et al., 2011; Yamamoto and Yue, 2014), the impairment of autophagy will result in accumulation of aggregated proteins and damaged organelles, which are common pathological hallmarks in AD and PD. Accumulating evidence indicates that the autophagy machinery in microglia can contribute to the emergence, acceleration, or amelioration of CNS disease conditions (Keller et al., 2020). So far, two specific mechanisms appear to be relevant to CNS pathology: activation of the inflammasome and increase of autophagy protein-mediated endocytosis/phagocytosis. Autophagy pathways are implicated in the regulation of inflammasome function at various steps by removing triggering agents, inflammasome constituents, or downstream effector molecules (Saitoh et al., 2008; Harris et al., 2011; Liu et al., 2016). As the major cellular component of the innate immune system in the brain, microglia have been found to execute pivotal functions during CNS homeostasis and pathology (Heneka et al., 2014, 2018).

In response to microenvironmental stimuli and factors, microglia can change morphologically and functionally to migrate to the injury sites. They can recognize harmful entities through Toll-like receptors (TLRs) and trigger receptor expression in myeloid cells 2 (TREM2; Blander and Medzhitov, 2004; Takahashi et al., 2005) and signaling pathway activation that leads to reorganization of new phagosomes (Koizumi et al., 2007; Yao et al., 2019). Canonical autophagy pathways require a multistep assembly mechanism. In most cells, ATG proteins regulate the formation of the autophagosome that involves multiple proteins and molecular changes. Initiation of autophagy requires the association of the unc-51-like kinase 1 (ULK1) kinase complex (Shang and Wang, 2011), the phosphorylation of the transmembrane protein ATG9 and the class III phosphatidylinositol 3-kinase (PI3KC3) complex (Young et al., 2006; Russell et al., 2013; Zhou et al., 2017), the recruitment of phosphatidylinositol 3-phosphate (PI3P) effector proteins, and the conjugation of ubiquitin-like human ATG8 to phosphatidylethanolamine (PE) on a nascent phagophore (Dooley et al., 2014). Finally, the mature autophagosome fuses with lysosomes to autolysosomes, resulting in the degradation of cargos (Julg et al., 2020). So far, this kind of autophagy pathway has been found in many important cellular functions, including pathogen defense (Gutierrez et al., 2004; Ogawa et al., 2005), pro-inflammatory response (Saitoh et al., 2008; Houtman et al., 2019), and intracellular aggregates (Choi et al., 2020). In noncanonical autophagy processes, autophagy processes can be deployed to fulfill functions without involving lysosomal delivery of cytosolic cargo. For example, BECN1 is required for conjugating LC3 to the phagosomes or endosomes during this process, and the phagocytic ingestion and subsequent degradation of Aβ plaques (Sanjuan et al., 2007; Heckmann et al., 2019). Deletion of BECN1 in BV2 microglial cells in APP transgenic mouse brain slices resulted in insufficient phagocytosis of Aβ (Lucin et al., 2013). In the meantime, microglia from human AD brains exhibit reduced BECN1 (Lucin et al., 2013). So far, studies related to the canonical autophagy pathways have been widely reported in microglia, while studies related to the noncanonical process still need to be further explored.

### Microglia engulf apoptotic cells during degeneration

Given that microglia are resident immune cells, one of their roles is to ingest and clear neuronal debris resulting from programmed cell death during CNS development, a process that constantly senses the neural environment and eliminates excess neurons generated as part of normal neurogenesis in postnatal development and adult (Nimmerjahn et al., 2005; Marin-Teva et al., 2011; Brown and Neher, 2014). Microglia are not only the debris cleaner activated by damaged or dying neurons, but also are active neuronal scavengers to drive neuronal programmed cell death by inducing apoptosis. Neuronal apoptosis would be induced by the release of nerve growth factor (Frade and Barde, 1998), tumor necrosis factor (Sedel et al., 2004; Takahashi et al., 2005), glutamate (Bessis et al., 2007), or superoxide ions (Marin-Teva et al., 2004; Wakselman et al., 2008) from microglia. In the innate immune system, complement proteins function as “eat me” signals, which can mark apoptotic cells for removal by macrophages that express C3 receptors (CR3; van Lookeren Campagne et al., 2007). And microglia are the only CNS cell type that expresses CR3 (Schafer et al., 2012). Under physiological conditions, the “blinded” microglial phagocytosis is coupled to apoptosis through “find-me” signals, such as UDP and ATP, released by apoptotic cells (Elliott et al., 2009; Abiega et al., 2016).

However, phagocytosis is impaired during aging (Gabande-Rodriguez et al., 2020), and blocking phosphatidylserine (PS) with Annexin V decreases almost all of the capacity of microglial phagocytosis of damaged neurons (Krasemann et al., 2017). This will increase the number of phagocytosing microglia, a mechanism that could be targeted in pathological conditions (Abiega et al., 2016; Salter and Stevens, 2017). Thus, uncoupling of phagocytosis will lead to an accumulation of apoptotic cells and build up a detrimental microenvironment.

### Dynamics of microglia in responding to degeneration

In the CNS, one of the main cell types that are responsible for removing aggregated proteins from brain parenchyma is microglia. Microglia continuously supervise and process the microenvironment in search of stimuli or inflammatory signals. Microglia respond to numerous signals such as amyloid-beta (Aβ) peptides (Zhang et al., 2011), α-synuclein (α-syn) (Zhang et al., 2007), complement, and cytokines (Aloisi, 2001). Through this surveillance, they detect diverse extracellular signals, and consequently integrate with and respond to microenvironmental alterations to maintain CNS homeostasis (Salter and Stevens, 2017).

Microglia contribute to the clearance of Aβ peptides by phagocytosis and the degradation process by releasing enzymes, such as insulin-degrading enzyme (IDE), which can degrade Aβ in the extracellular space (Tamboli et al., 2010; Heneka et al., 2015). Several microglia surface receptors have been shown to mediate phagocytic clearance of Aβ peptides, including TLR2, TLR4, and TLR6 and CD14, CD36, and CD45 (Grommes et al., 2008; Zhu et al., 2011). At the same time, the delivery process of Aβ is also pivotal. For example, neuronal exosomes can bind Aβ peptides and accelerate its phagocytic clearance by microglia in a phosphatidylserine-dependent way (Yuyama et al., 2012). Microglia can phagocytose and clear extracellularly aggregated α-syn faster than other cells (Lee et al., 2008a). Also, autophagy protects the nervous system by suppressing NLRP3 inflammasome activation (Su et al., 2016). Deletion of DJ1 leads to impaired microglial autophagy and reduces the ability of microglia to uptake and degrade extracellular α-syn, which could exacerbate the pro-inflammatory situation in DJ1 KD microglia (Nash et al., 2017). Therefore, exploring the detailed molecular mechanism of microglial autophagy is crucial for the cure of neurodegeneration.

### The autophagy of microglia in Alzheimer’s disease

Alzheimer’s Disease is the most prevalent neurodegenerative disease in the world, affecting 55 million people by 2021, and is expected to reach 78 million by 2030. The most obvious pathological features of AD patients are intracellular Tau tangles and extracellular Aβ plaques. In the late stage of human AD, a plenty of neurons are lost in the cortex and hippocampus (Nelson et al., 2012). At present, there have been many studies about the mechanism of AD, among which neuroinflammation is considered to be an important factor in the pathogenesis of AD. Therefore, it is necessary to explore the autophagy function of microglia in AD.

## Abnormal Aβ and Tau can be pathogens in AD

During the development of AD, Aβ plaques form in the brain of patients as a result of accumulation of Aβ peptides resulting from the cleavage of amyloid precursor protein (APP) by γ-secretase and β-site APP cleaving enzyme 1 (Hampel et al., 2020). In AD brain, alterations in AMPK signaling are a central issue (Vingtdeux et al., 2010; Zimmermann et al., 2020). As a homeostasis sensor, AMPK activates microglial autophagy in the presence of Aβ, consequently leading to their lysosomal degradation (Cho et al., 2014; Julg et al., 2020). The activated microglial cells are predominantly located surrounding Aβ plaques (Frautschy et al., 1998; Nicoll et al., 2003; Zotova et al., 2013). Many studies have found that the degradation of Aβ plaques is accomplished through the process of autophagy in microglia (Lee and Landreth, 2010; Kitazawa et al., 2011). Activation of peroxisome proliferator-activated receptor-α (PPARA)-mediated induction of microglial autophagy with gemfibrozil and Wy14643 has been associated with amelioration of AD-like phenotype in the APP-PSEN1 mouse model (Luo et al., 2020). Also, disruption of noncanonical autophagy in microglia *via* targeted deletion of Rubicon and *atg5 in vivo* leads to the increased deposition of toxic Aβ and subsequent cognitive impairment in the 5 × FAD AD mouse model (Heckmann et al., 2019). Estfanous et al. reported that the ability of microglia to degrade Aβ was inhibited in AD patients compared with normal people (Estfanous et al., 2022). ACAT1/SOAT1 inhibitor K604 can improve the ability of microglia to clear Aβ-42 (Shibuya et al., 2014). These studies demonstrate that microglia can clear Aβ through autophagy process.

In AD patients, another pathological feature is neurofibrillary tangle that is formed by aggregation of abnormal phosphorylation Tau protein, which results in dysfunction of Tau protein, decrease in microtubule stability, and loss of neuronal function (Alonso et al., 2018). Hyperphosphorylated Tau protein in AD patients can be secreted into the extracellular part of neurons (Fiandaca et al., 2015; Jia et al., 2019), and microglia could engulf oligomers of extracellular Tau (Majerova et al., 2014). Other studies have also illustrated that Tau can activate microglia to promote brain inflammation (Jin et al., 2021). Zilkova et al. found that Tau antibodies could promote the absorption of Tau in human microglia (Zilkova et al., 2020). Furthermore, many studies have shown that activated microglia are involved in Tau-mediated lesions, such as Tau aggregation (Gorlovoy et al., 2009; Maphis et al., 2015), transmission (Asai et al., 2015; Wang et al., 2022), and Tau phagocytosis (Brelstaff et al., 2018; Hopp et al., 2018). Researchers have found that inhibition of microglia proliferation can improve Tau-induced neuronal degeneration and loss of function (Mancuso et al., 2019). These studies suggest that Tau and microglia have mutually reinforcing effects.

## Microglia recognize pathogens by pattern recognition receptors

As mentioned above, both extracellular Aβ aggregation and Tau protein secretion can cause changes in microglia, but how these abnormal proteins activate microglia remains unclear. Under normal physiological conditions, microglia protect the CNS by reducing harmful stimuli, such as pathogenic molecular patterns (PAMPs) and damaging molecular patterns (DAMPs). Some receptors, such as TLRs and nuclear oligomerization domain-like receptors, and viral receptors, are expressed on the surface of microglia. All these receptors belong to pattern recognition receptors (PRRs) and can recognize PAMPs and DAMPs, thus leading to the activation of microglia (Glass et al., 2010). Similarly, Aβ and tau can be recognized by microglial PRRs, such as TLR2, TLR4, cluster of differentiation 14 (CD14), and cluster of differentiation 47 (CD47). Upon binding to these receptors, Aβ and tau can be internalized to induce inflammation through specific pathways involving NLRP3, MYD88, or NF-κB (Taro and Shizuo, 2007; Dansokho and Heneka, 2018; Kabzinski et al., 2019; Lee J. H. et al., 2021; Leng and Edison, 2021), leading to the transcription of pro-IL-β and NLRP3 (Lee J. H. et al., 2021; Leng and Edison, 2021) and the phagocytic activity of microglia. Stimulated and activated microglia also produce pro-inflammatory cytokines, such as tumor necrosis factor-α (TNF-α), interleukin-1β (IL-1β), IL-16, and chemokines (Kabzinski et al., 2019). In turn, phagocytosis removes pathological stimuli to maintain brain homeostasis.

PRRs-mediated signal transduction promotes the release of inflammatory cytokines from microglia. TLR is expressed in cell membranes and the cytoplasm, and the TLR in cytoplasm can detect the nucleic acid of viruses and bacteria. Some TLRs can bind to other molecules, such as CD14 and CD36, as co-receptors (Dansokho and Heneka, 2018).

**Figure 1:**
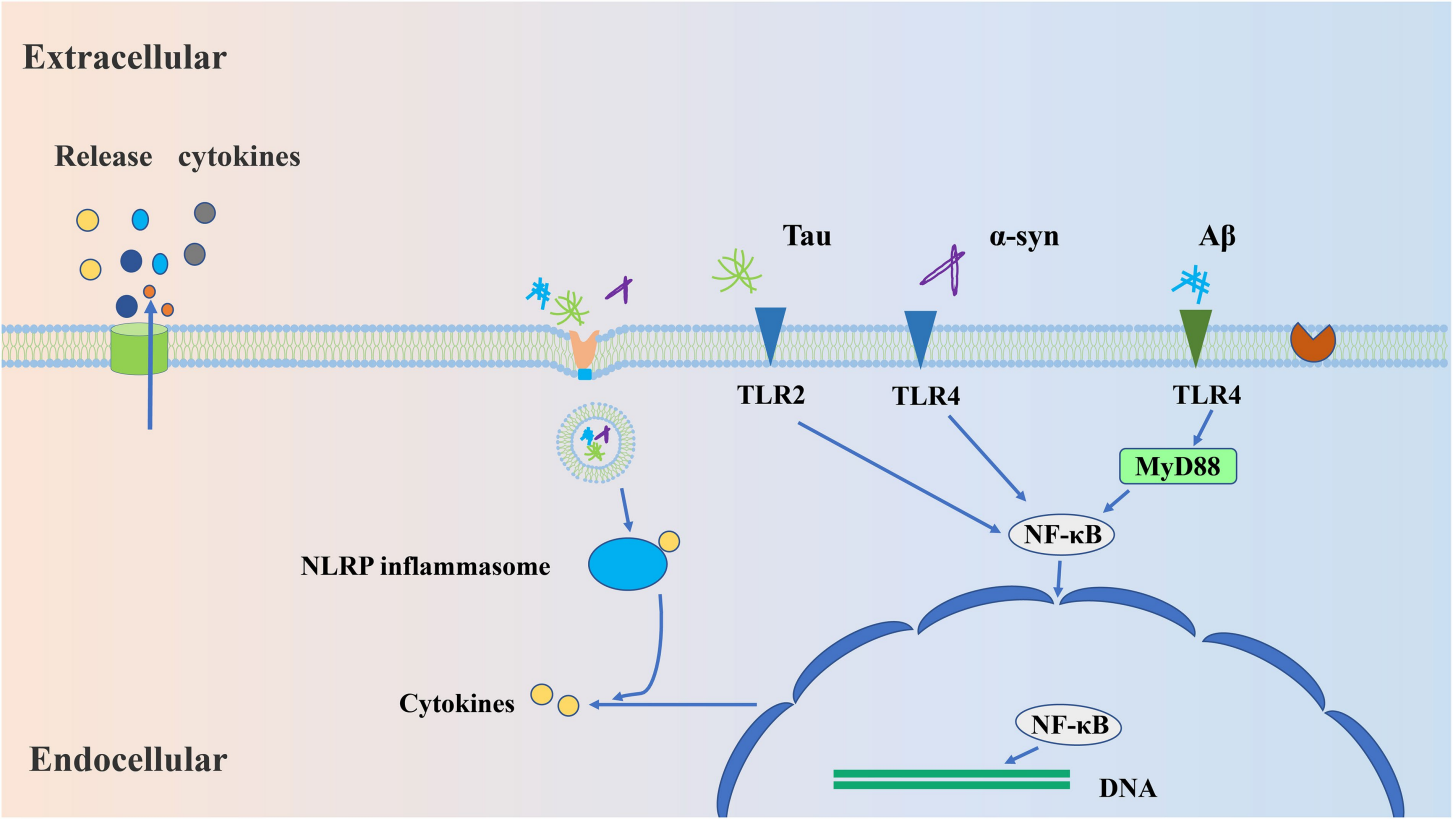
The mechanisms by which microglia respond to aggregated proteins. Firstly, aggregates (Aβ/Tau/α-syn) are recognized by microglial pattern recognition receptors, such as TLR2 and TLR4. Afterward, these receptor binding activates the specific signaling pathways involving MYD88/NF-κB, which leads to transcription of NLRP3 and subsequent enables the release of inflammatory cytokines, namely IL-18 and IL-β. Further, the release of cytokines will induce chronic inflammatory responses.

TLR-mediated signaling pathways lead to the production of type I IFNs and pro-inflammatory cytokines from microglia (Figure 1), and there are two ways to contribute to the release of inflammatory cytokines, MyD88 and TRIF-dependent pathways, respectively (Taro and Shizuo, 2007). It has pointed out that the activation of TRL4 produces beneficial (Michaud et al., 2013) and harmful (Zhang et al., 2015) inflammatory reactions in AD. TLR2 has been reported to produce a severe inflammatory response to Aβ (Nixon et al., 2005). However, activation of TRL9 facilitates the clearance of Aβ (Scholtzova et al., 2014). These studies suggest that activation of different TLRs produces different inflammatory responses, and that the same TLR also produces different inflammatory responses to different stimuli. It has also been shown that the activation of TLR2 and TLR4 in the early stage of AD contributes to the clearance of Aβ, while long-term activation may lead to the accumulation of Aβ (Lee S. et al., 2021). Therefore, the inflammatory response mediated by TLRs is complex, and we need to further understand the role of different TLRs in the signaling pathways.

Inflammasomes are a group of polyprotein complexes, which exist in microglia and can also recognize PAMPs or DAMPs. Therefore, inflammasomes are also considered to belong to intracellular PRRs-NOD-like receptors (NLRs) (Strowig et al., 2012). Inflammasome has various forms, including NLRP1, NLRP3, NLRP6, NLRP7, NLRP12, etc. (Dansokho and Heneka, 2018). Under normal physiological condition, the NLRP inflammasome forms vesicle-autophagosome with PAMPs or DAMPs, which then fuses with lysosome resulting in degradation of the cargo. Nevertheless, excessive activation of NLRP3 inflammasome can contribute to development of inflammatory diseases (Biasizzo and Kopitar-Jerala, 2020). NLRP inflammasome plays a certain role in the pathogenesis of AD. In monocytes of AD patients, the mRNA and protein levels of NLRP3 are significantly increased (Saresella et al., 2016), and NLRP1 is activated in the hippocampus of AD patients (Španić et al., 2022). The abnormal Aβ and Tau induce inflammation through specific pathways involving MYD88 or NF-κB (Yang et al., 2020; Barczuk et al., 2022), leading to the transcription of pro-IL-β and NLRP3 (Lee J. H. et al., 2021; Leng and Edison, 2021). Increasing evidence has shown that soluble Aβ oligomers can activate the NLRP3 inflammasome in microglia (Lučiūnaitė et al., 2020), while deletion or inhibition of NLRP3 in APP/PS1 in mice will reduce spatial memory loss and Aβ aggregation (Michael et al., 2012; Dempsey et al., 2017; Feng et al., 2018). In addition, p-Tau and aggregated Tau can also activate the NLRP3 inflammasome in microglia and further aggravate Tau lesions (Jiang et al., 2021). Similarly, inhibition of NLRP3 in TauP301S transgenic mice reduces Tau phosphorylation and Aβ accumulation in the hippocampus (Stancu et al., 2019). In conclusion, these studies show that NLRP3 inflammasome plays an important role in the development of AD, perhaps because the excess NLRP3 inflammasome in AD cannot be degraded by autophagy, leading to a more severe inflammatory response.

### The autophagy of microglia in PD

PD is the second most common neurodegenerative disease after AD. The main pathological features of PD patients consist of the loss of dopaminergic neurons (DA) neurons in the SN pars compacta and the abnormal aggregation of α-syn in the form of Lewy bodies. Typical clinical features of PD include bradykinesia, tremor, muscle rigidity, and postural instability. Activated microglia, as well as high levels of reactive oxygen species (ROS) and many inflammatory factors such as TNF-α and IL-1β, were observed in both the SN and striatum of PD patients (McGeer et al., 1988; Mogi et al., 1994a,b). Many articles have shown that the loss of microglial autophagy exacerbates the inflammatory response, affects the survival of neurons, and accelerates the progression of PD (Su et al., 2016; Nash et al., 2017; Houtman et al., 2019; Qin et al., 2021; Cai et al., 2022). However, the mechanisms linking dopaminergic neuronal death to microglial autophagy have not been completely elucidated.

### NLPR3 inflammasome and microglial autophagy

Microglial autophagy and inflammatory response are necessary for protecting against external stimuli. When the inflammatory response in the brain is continuously activated, overactivated inflammasomes can cause neuronal damage (Lee et al., 2019; Ji et al., 2020). As early as 2006, it was reported that neuronal autophagy dysfunction induces neurodegenerative diseases in mice (Komatsu et al., 2006), and recent studies have linked microglial autophagy to NLRP3 inflammasomes (Su et al., 2016; Biasizzo and Kopitar-Jerala, 2020; Badanjak et al., 2021), elucidating the important role of NLRP3 inflammasomes activation triggered by autophagy deficiency in microglial cells in the development of PD.

NLR family pyrin domain 3 (NLRP3), a widely studied oligomeric multiprotein inflammasome complex, is highly expressed in microglia. Microglial hyperactivation of the NLRP3 inflammasome has been well-documented in various neurodegenerative diseases, including PD (Lee et al., 2019; Wang et al., 2019; Biasizzo and Kopitar-Jerala, 2020; Ahmed et al., 2021). Autophagy protects the nervous system by clearing NLRP3 inflammasome activation (Su et al., 2016; Cai et al., 2022). Likewise, inflammasome signaling pathways can also regulate microglia activation necessary to balance between required host defense inflammatory response and to prevent excessive and detrimental inflammation (Lee et al., 2008b; Freeman et al., 2017).

Deletion of an *atg5* in microglia causes age-dependent PD-like symptoms in mice (Cheng et al., 2020; Tu et al., 2021). Microglia-specific knockout of *atg5* had no obvious PD symptoms at 10 weeks of age in mice (Qin et al., 2021); however, there is a marked PD-like impairment of motor coordination in mice as young as 3 months old due to excessive inflammasome-mediated release IL-1β and subsequent production of pro-inflammatory factor (Cheng et al., 2020), implying that the autophagy damage caused by *atg5* was age-dependent. Impaired autophagy in microglia may result in excessive NLRP3 inflammasome activation, which may aggravate MPTP-induced dopaminergic neuronal injury and neuroinflammation in PD mice (Qin et al., 2021). And inhibition of NLRP3 inflammasome activation by administration of the NLRP3-specific inhibitor MCC950 reduced neuroinflammation levels and rescued the loss of tyrosine hydroxylase (TH)-positive neurons in the SN (Cheng et al., 2020). The mechanism by which impaired autophagy resulted in enhanced NLRP3 activation in the context of PD is unclear, but NLRP3 inflammasome activity is negatively regulated by microglial autophagy (Plaza-Zabala et al., 2017; Houtman et al., 2019; Qin et al., 2021). In conclusion, the role of autophagy in microglia has been identified during the development of PD. Neuroinflammation is a critical initiation step for dopaminergic neuron degeneration and PD development. Microglial autophagy deficiency may sensitize the cells to stimulation and boost neuroinflammation (Qin et al., 2021; Xu et al., 2021). Targeting microglial autophagy might be an effective way to regulate neuroinflammation in the treatment of neurodegenerative diseases (Houtman et al., 2019; Bartels et al., 2020; Cheng et al., 2020).

Downregulation of DJ1, a PD-related protein, exacerbates neuroinflammation and oxidative stress (Taira et al., 2004; Nash et al., 2017). Knockdown of DJ1 *via* Nrf2/Trx1/NLRP3 axis accelerated microglia-mediated neuroinflammation and apoptosis (Ji et al., 2020). In addition, the relationship between LRRK2, another important PD-related protein, and autophagy and neuroinflammation has been extensively studied. We can learn more about the relationship between LRRK2 and microglial autophagy from a recently published review (Zhang et al., 2022).

### α-syn and microglial autophagy

In PD, neuron-released α-syn and its accumulation in Lewy bodies result in degeneration of the SN dopaminergic system (Xu et al., 2002). Oligomeric α-syn secreted by neurons is toxic and can mediate the activation of microglia through TLRs (Figure 1), especially TLR2 and TLR4 mediates (Fellner et al., 2013; Kim et al., 2013), and therefore induces a chronic inflammatory response (Lee et al., 2008a; Béraud and Maguire-Zeiss, 2012; Choi et al., 2020). Intriguingly, impaired microglial autophagy provokes a decrease of DA when α-syn is expressed in mice (Choi et al., 2020). Deletion of PD-associated DJ1 protein leads to impaired microglial autophagy, reduces the ability of microglia to uptake and degrade extracellular α-syn, and exacerbates pro-inflammatory in DJ1 knockdown microglia (Nash et al., 2017). Although glia and neurons in the brain can take in and degrade extracellular α-syn, microglia show the highest efficiency through selective autophagy *in vitro* and *in vivo* (Lee et al., 2008a; Choi et al., 2020). But excess α-syn also inhibits microglial autophagy and exacerbates oxidative stress. Blocking Drp-1 would enhance autophagy flux and inhibit α-syn aggregation to reduce exosome release (Fan et al., 2019). Therefore, clearing extracellular α-syn by microglial autophagy is considered to be a vital way in maintaining neuronal function and is crucial for the treatment of PD.

**Figure 2:**
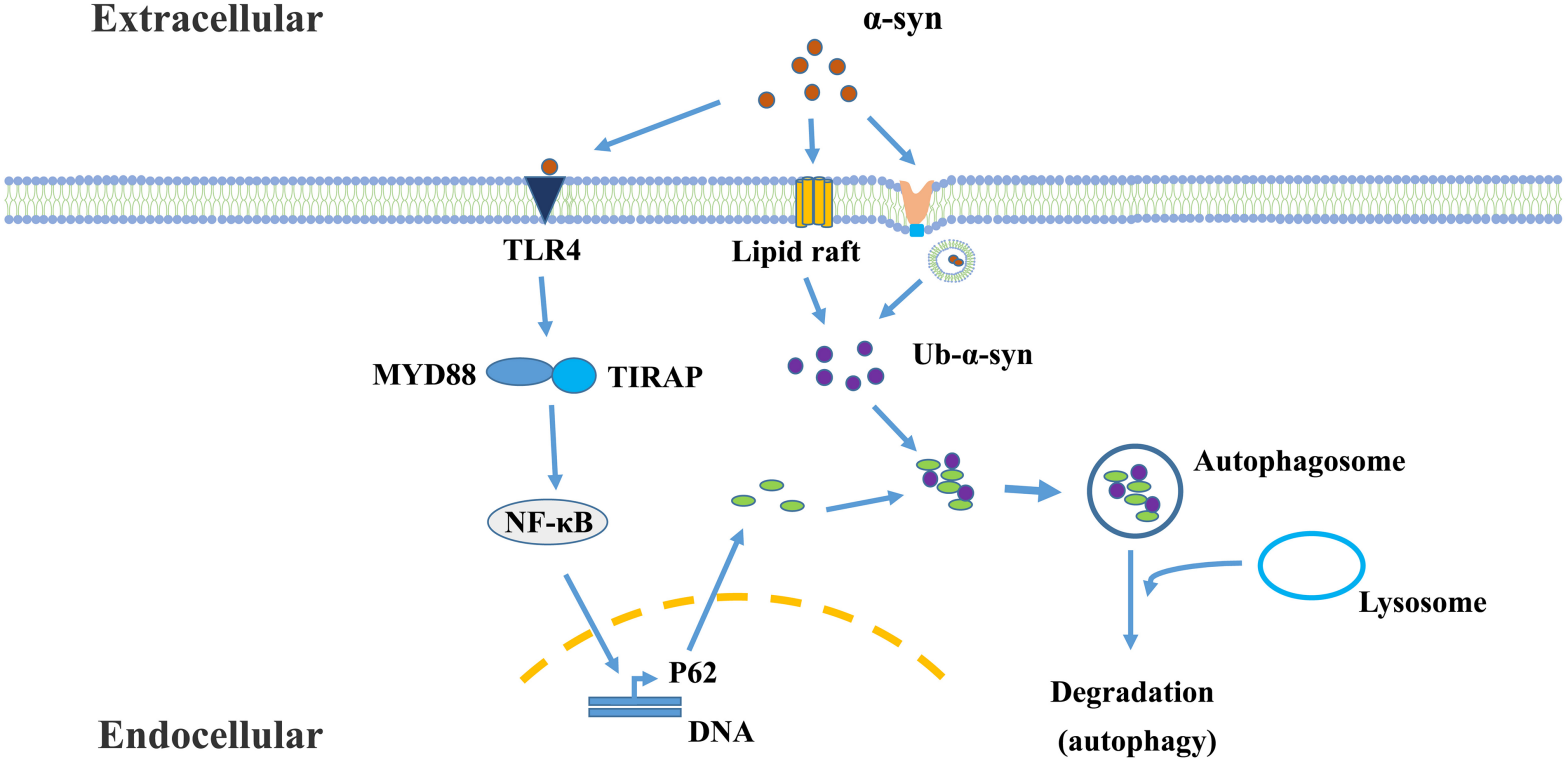
The mechanism of microglial synucleinphagy. Microglia ingest extracellular α-syn protein, which is sequestered by the autophagy-lysosome pathway. This process is mediated through TLR4-NF-κB signaling, and leads to the endocytosis- and phago-independent ingestion of extracellular α-syn. In this process, α-syn-TLR4 interaction stimulates p62 expression mediated by NF-κB. Meanwhile, α-syn can enter the cells or penetrate cytoplasmic membrane through other endocytosis-independent processes [such as lipid raft (Park et al., 2009)] or endocytosis-dependent processes (Lee et al., 2008a). Then, oligomeric p62 binds and recruits ubiquitinated-α-Syn (Ub-α-Syn) into autophagosomes for degradation.

Recently, a cargo-selective autophagy process in microglia, termed “synucleinphagy” (Figure 2), has been identified to promote neuroprotection by efficient clearance of neuron-derived α-syn in a PD mouse model (Choi et al., 2020). This process is mediated through TLR4-NF-κB signaling by transcriptional upregulation of the autophagy receptor, p62/SQSTM1, and leads to the endocytosis- and phago-independent ingestion of extracellular α-syn. At last, the cargo will be targeted into autophagosomes. It is assumed that this binding is mediated by p62’s recognition of ubiquitinated α-syn (Kuusisto et al., 2001; Lamark et al., 2009; Julg et al., 2020). Future studies should identify the specific E3 ligase of α-synuclein in microglia responsible for synucleinphagy. However, neurons do not degrade α-syn through this pathway due to the lack of the TLR4-NF-κB pathway (Redmann et al., 2017; Choi et al., 2020). The question of how α-syn is taken up by microglia and released into the cytosol remains to be addressed. Studies have shown that the ability of microglia to selectively clear α-syn by autophagy is important for alleviating neuronal damage. It has been confirmed that extracellular α-syn induces autophagy damage in microglia by activating TLR4 and its downstream P38 and AKT/mTOR signaling (Tu et al., 2021), affects the sensitivity of microglia to senescence, and accelerates the evolvement of PD. Extracellular pathological α-syn exacerbates dopamine neuronal loss and reduced lysosomal activity of microglia in TLR4-deficient mice (Venezia et al., 2021). α-syn binds to TLRs on the surface of microglia, and different types of α-syn may interact with different autophagy proteins in microglia, which determines whether α-syn is degraded by microglia or inhibits microglial autophagy.

### Therapeutic opportunities by targeting microglia

Targeting microglial autophagy has been proposed to have great therapeutic potential in these diseases (Uddin et al., 2018). In neurodegenerative diseases, the mitochondria in microglia can be damaged by the misfolded protein aggregations and released mtDNA and ROS, which over-activates the NLRP3 inflammasome. Autophagy inducers can decrease the levels of mtDNA and ROS *via* the autophagic degradation of damaged mitochondria (Wu et al., 2021). The enhancement of autophagy by inducers to inhibit the NLRP3 inflammasome-mediated inflammatory responses through the degradation of the damaged mitochondria and generated ROS (Gurung et al., 2015; Dai et al., 2017), is a promising strategy. Recently, many new drugs, like nanoparticles, which can cross the blood–brain barrier, have brought light to the treatment of AD and PD.

#### AD treatment

Currently, Aβ accumulation indicates the initial effect that drives Tau-seeded pathologies and Tau-mediated neurotoxicity and pathogenesis in AD (He et al., 2018). Anti-Aβ antibody and nanoparticles-mediated Aβ clearance have been the mainstream direction of anti-AD drug development (Fernandez-de-Retana et al., 2017). Aducanumab, a monoclonal antibody, has been approved for the treatment of AD, which selectively targets aggregated Aβ (Sevigny et al., 2016) and promotes Aβ clearance in the brain. But monoclonal antibodies against Tau are still in the early experimental stage at present (Ji and Sigurdsson, 2021). The latest report shows us a “Drug-Carrier” synergy therapy, which is designed to simultaneously target Aβ and Tau-associated pathways for AD treatment (Han et al., 2022). In this system, the endogenous apolipoprotein A-I and its mimicking peptide 4F fused angiopep-2 are sequentially grafted onto lipid nanocomposite, which can provide the liberty of blood–brain barrier crossing and microglia targeted Aβ clearance. For synergy treatment, methylene blue is further assembled into APLN for Tau aggregation inhibition. After the treatment with “Drug-Carrier” synergy therapy in AD-Aβ-Tau bearing mouse models, this treatment rescued neuron viability and cognitive functions.

#### PD treatment

Andrographolide (Andro), a bicyclic diterpenoid lactone, has been reported to exhibit immunomodulatory, anti-inflammatory, and anti-viral (Panraksa et al., 2017; Ahmed et al., 2021). Treatment with Andro promoted the parkin-dependent autophagic flux formation in microglia, leading to the removal of defective mitochondria thereby inhibiting the activation of the NLRP3 inflammasome. Furthermore, Andro can rescue the loss of DA (Ahmed et al., 2021). Quercetin (3,3′,4′,5,7-pentahydroxyflavone; Qu), is one of the most common plant flavonoids and prominent dietary antioxidants in the human diet (Boots et al., 2008). Qu inhibits LPS-induced NLRP3 inflammasome assembly in BV2 cells and alleviates neuronal damage by promoting mitophagy, reducing mtROS accumulation. Qu treatment protected primary neurons from LPS-induced microglial toxicity and attenuated neurodegeneration in PD mice (Han et al., 2021). Recently, Yuan et al. constructed Cu_2-x_Se-anti-TRPV1 nanoparticles (CS-AT NPs), which can target microglia and open their surface TRPV1 channels, causing Ca^2+^ influx to activate ATG5 and Ca^2+^/CaMKK2/AMPK/mTOR signaling pathways and promoting autophagy-mediated clearance of α-syn PFFs by microglia (Yuan et al., 2022). Altogether, blocking microglia-mediated neuroinflammation and promoting mitophagy is a protective mechanism in halting the early progression of PD. Combining the use of the specific inhibitors of the NLRP3 inflammasome with autophagy inducers is more effective than one single treatment in cellular or animal models of neurodegenerative diseases (Chen et al., 2019; Xu et al., 2019).

## Conclusion

Autophagy and its dysfunction are associated with a variety of human pathologies, including aging, neurodegenerative disease, heart disease, cancer, and metabolic diseases, such as diabetes. A plenty of drugs and natural products have been found to modulate autophagy function through multiple signaling pathways. Small molecules or nanomedicine that can regulate autophagy seem to have great potential to intervene in neurodegenerative diseases that are largely due to the accumulation of misfolded proteins. Results from the present study indicate a correlation between microglial autophagy capacity and severity of neurodegeneration, and also provide important resources to better understand the pathogenesis of these important brain diseases. However, there are a few issues that need to be paid attention to. First, because autophagy function is impaired by aging (Gabande-Rodriguez et al., 2020), it is necessary to improve autophagy capacity in the aged brain. Second, it is also important to increase the efficiency of drug to cross the blood–brain barrier. Last but not the least, a deeper understanding and accurate detection of the early pathological changes in neurodegenerative diseases is important for developing more effective therapeutic methods.

## Author contributions

All authors listed have made a substantial, direct, and intellectual contribution to the work and approved it for publication.

## Funding

The research presented in this article was supported by National Key Research and Development Program of China (2021YFF0702201), Natural Science Foundation of Guangdong Province (2022A1515012651, 2022A1515012301), the National Natural Science Foundation of China (32070534, 81830032, and 31872779), Guangzhou Key Research Program on Brain Science (202007030008), and Department of Science and Technology of Guangdong Province (2021ZT09Y007 and 2020B121201006).

## Conflict of interest

The authors declare that the research was conducted in the absence of any commercial or financial relationships that could be construed as a potential conflict of interest.

## Publisher’s note

All claims expressed in this article are solely those of the authors and do not necessarily represent those of their affiliated organizations, or those of the publisher, the editors and the reviewers. Any product that may be evaluated in this article, or claim that may be made by its manufacturer, is not guaranteed or endorsed by the publisher.
